# Effects of a movement control and tactile acuity training in patients with nonspecific chronic low back pain and control impairment – a randomised controlled pilot study

**DOI:** 10.1186/s12891-020-03727-y

**Published:** 2020-11-30

**Authors:** Katharina van Baal, Jana Allofs, Katja Ehrenbrusthoff, Christian Grüneberg, Thomas Hering, Christian Kopkow, Christian Thiel

**Affiliations:** 1 Department of Applied Health Sciences, University of Applied Sciences Bochum, Gesundheitscampus 6–8, 44801 Bochum, Germany; 2 Institute for General Practice, Hannover Medical School, Carl-Neuberg-Straße 1, 30625 Hannover, Germany; 3 Department of Applied Human Sciences, University of Applied Sciences Magdeburg-Stendal, Osterburger Str. 25, 39576 Stendal, Germany; 4 Department of Applied Therapy Sciences, Brandenburg University of Technology Cottbus-Senftenberg, Universitätsplatz 1, 01968 Senftenberg, Germany

**Keywords:** Chronic low back pain, Physical therapy modalities, Tactile acuity, Motor control, Low back pain, Feasibility

## Abstract

**Background:**

Nonspecific chronic low back pain (NSCLBP) is a heterogeneous condition that is associated with complex neuromuscular adaptations. Exercise is a widely administered treatment, but its effects are small to moderate. Tailoring patient-specific exercise treatments based on subgroup classification may improve patient outcomes.

**Objective:**

In this randomised controlled pilot study, our objective was to compare the feasibility and possible effects of a specific sensorimotor treatment (SMT) with those of a general exercise (GE) programme on patients with NSCLBP and control impairment (CI).

**Methods:**

Patients with NSCLBP and CI were randomised into an SMT or a GE programme spanning 6 sessions each. The feasibility criteria included the study design, assessments, interventions and magnitudes of effects, and costs. Adverse events were documented. Primary (pain, physical function, and quality of life) and secondary outcomes were assessed three times: twice at baseline (t1a and t1b) to estimate parameter stability and once after the intervention (t2).

**Results:**

Two-hundred and twenty-seven patients were screened to include 34 participants with NSCLBP and CI. Both treatment programmes and the assessments seemed feasible because their durations and contents were perceived as adequate. The total cost per participant was €321. Two adverse events occurred (one not likely related to the SMT, one likely related to the GE intervention).

The SMT showed a tendency for superior effects in terms of pain severity (SMT t1a 3.5, t2 1.1; GE t1a 3.0, t2 2.0), pain interference (SMT t1a 1.9, t2 0.4; GE t1a 1.5, t2 0.9), physical component of quality of life (SMT t1a 39, t2 46; GE t1a 45, t2 48), and movement control.

**Conclusions:**

The SMT approach proposed in this study is feasible and should be tested thoroughly in future studies, possibly as an addition to GE. To ensure the detection of differences in pain severity between SMT and GE in patients with NSCLBP with 80% power, future studies should include 110 patients. If the current results are confirmed, SMT should be considered in interventions for patients with NSCLBP and CI.

**Trial registration:**

Registered in the German Register for Clinical Trials (Trial registration date: November 11, 2016; Trial registration number: DRKS00011063; URL of trial registry record); retrospectively registered.

## Background

Chronic low back pain (CLBP) is a major social problem in Western societies with a prevalence of 4.2 to 19.6% [1]. A large proportion of patients with CLBP present without clear cause and pathoanatomical correlation to their symptoms [2, 3]. These patients are, therefore, classified as having nonspecific chronic low back pain (NSCLBP) [2–5].

A number of interventions, for example, exercise therapy, pharmacological approaches, back schools, progressive relaxation, and multidisciplinary treatment approaches, have been tested in clinical studies involving patients with NSCLBP, and they mostly induced small to moderate effects on pain and physical function [6–9]. Current guidelines advocate a multimodal conservative treatment [8, 9]. This biopsychosocial approach involves a combination of pain education, cognitive behavioural therapy, relaxation methods, physical exercise, risk factor management, and supportive pharmacotherapy. Exercise therapy and staying physically active are often considered core elements of conservative treatment, but there is little evidence for the general superiority of the individual components or exercise modes [6].

Patients with NSCLBP exhibit various clinical patterns [10, 11]. Thus, it has been suggested that instead of providing fixed sets and contents of treatments (the one-size-fits-all approach), it might be more appropriate to stratify treatment to subgroups or tailor the choice and dose of interventions towards individual clinical patterns [6, 12].

Indeed, clinicians and researchers have started to identify the subgroups that may benefit the most from a specific treatment [13–15]. The STarT (Subgroups for Targeted Treatment) Back Screening Tool has attracted considerable attention in recent years and can be used, among other tools, to create subgroups of patients with NSCLBP [16, 17]. Furthermore, O’Sullivan et al. developed a CLBP classification system with sufficient inter-rater reliability and validity [11, 18–21]. Based on clinical patterns, this system classifies patients into three subgroups [11]:

1. Adaptive pain behaviour: Patients show movement and/or control impairment (CI) as an adaptive response to the pathological processes that primarily drive the pain.
2. Centrally driven pain (no structural reason): Patients experience high levels of disability and pain, psychosocial features (such as fear, anger, and mal-adaptive coping), and changes in movement or motor control in response to the psychological and/or social factors that primarily drive the pain.
3. Mal-adaptive movement impairment or CI: Patients show maladaptive movement or control impairments that unconsciously drive the pain.

Patients belonging to any of the three subgroups may experience CI. Specifically, maladaptive movement or control impairments may possibly drive the state of symptoms in the third subgroup and act as the underlying mechanism, which opens up the possibility of treatment through targeted interventions [11].

It is believed that patients with CI adopt postures and movement patterns that stress their pain sensitive tissues without being aware that they are doing so [11, 22, 23]. Consequently, patients with CI may suffer from mechanically induced pain in static or dynamic postures and may perform aberrant movements without any active movement restriction. However, clear evidence is lacking that limited movement control contributes to the persistence of low back pain (LBP) in patients with CI [11, 19].

Several procedures for measuring lumbar movement control have been developed [11, 24, 25]. Luomajoki et al. [26] suggested a set of 6 clinical tests with substantial intra- and inter-rater reliability. Moreover, a degree of known-groups validity of this set of tests in terms of its ability to help testers to distinguish between patients with NSCLBP and healthy individuals has been demonstrated [26–28].

Few studies have explicitly addressed the effects of interventions that are tailored to NSCLBP subgroups. Vibe Fersum et al. [29] found that in patients with NSCLBP, classification-based cognitive, movement, and functional therapies had stronger effects than traditional manual therapy and exercise. Moreover, in patients with CI, specific movement control exercises have been found to be as effective and marginally more effective in reducing disability and improving function, respectively, than a general exercise (GE) programme [30, 31].

A growing body of evidence from brain imaging studies indicates that cortical reorganisation in specific brain areas, such as the primary somatosensory (S1) [29, 32–34] or motor cortex [35–40], may contribute to the persistence of symptoms in patients with NSCLBP. Interventions targeting the reversal of such cortical reorganisation have reduced complex regional pain syndrome (CRPS) and phantom limb pain [41–45]. Tactile acuity, as the current proxy measure of cortical reorganisation, was investigated in patients with CRPS and phantom limb pain. Patients with NSCLBP exhibited similar abnormalities in tactile acuity as those with CRPS and phantom limb pain [32]. Moreover, working body scheme, body perception, and motor control of the lumbar spine were altered in patients with NSCLBP [27, 28, 46–48].

There is scarce knowledge regarding the feasibility of specific sensorimotor treatments (SMTs), including motor control exercises and tactile acuity training, for the subgroup of patients with NSCLBP and CI. Therefore, a randomised controlled pilot study is needed.

In the present pilot study, we aim to evaluate the feasibility and estimate the effects of lumbar motor control exercises in combination with lumbar tactile acuity training in comparison to those of a general strengthening and coordination exercise training programme for patients with NSCLBP and CI.

## Methods

The study was a priori developed but retrospectively registered in the German Register for Clinical Trials (DRKS00011063) due to time constraints. Only the physical examination of potential participants was commenced before the final registration, while randomisation and intervention were commenced afterwards. Ethical approval was obtained from the ethics committee of the German Association of Physical Therapists (Deutscher Verband für Physiotherapie e. V., Ethics Committee No.: 2016–10).

To maintain the quality of reporting, we used the *CONSORT 2010 Statement: Extension to Randomised Pilot and Feasibility Trials guideline* [49] and the *Better Reporting of Interventions: Template for Intervention Description and Replication* (TIDieR) checklist [50].

### Study design

In this randomised controlled pilot study, the assessor was blinded to the intervention.

### Setting and participants

The participants were recruited from outpatient physical therapy practices in the Ruhr Metropolitan Area in Germany between September 2016 and February 2017. The recruitment was supplemented by distributing flyers by doctors’ offices, by students in a certificate course for manual therapy, through publication in regional newspapers, and through e-mails sent to the staff of the University of Applied Sciences, Bochum.

Patients were eligible for inclusion in the study if they met all of the following inclusion criteria:

- NSCLBP (duration ≥3 months) with or without leg pain
- fit into the CI subgroup
  - intermittent lower back pain
  - no restriction of the active range of movement in the painful direction
  - relation between pain and active movement (altered quality of movement)
- ≥18 years of age
- proficient in German language
- intact skin on the lower back

The exclusion criteria were as follows:

- serious pathology (such as fracture, tumour, and inflammatory disease)
- spinal surgery in the last year
- current or planned physical therapy treatment for the lower back or medical pain therapy over the study duration
- active exercise training with a movement control component more than twice a week
- acute neurologic symptoms, such as signs of nerve root compression (paraesthesia, weakness, and reflex changes) or symptoms distally of the knee worse than the LBP
- comorbid health conditions or pain prohibiting active exercise
- Roland-Morris Disability Questionnaire (RMDQ) scores > 18 [51]
- pregnancy

**Fig. 1:**
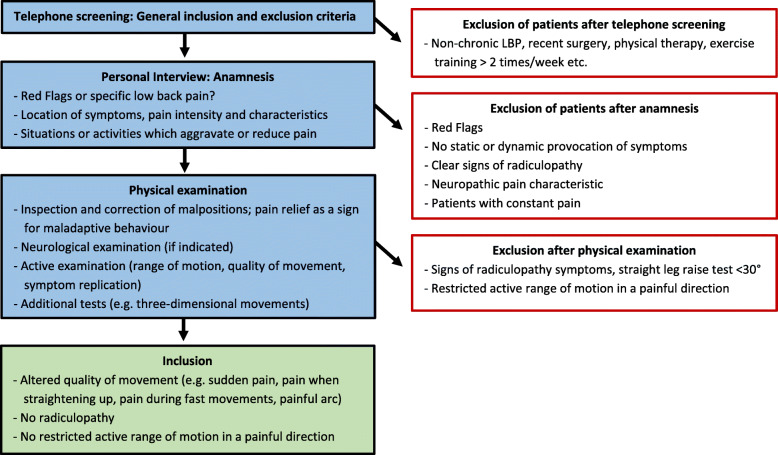
Process of screening and examination of potential participants to determine their eligibility

The complete process of screening and examination of potential participants is illustrated in Fig. 1.

### Randomisation and interventions

All eligible participants provided informed consent before they were included in the study. The participants were then allocated 1:1 to either a CI-specific SMT group or a GE group. The randomised lists of participants (blocks of 2 participants) were computer generated (SPSS version 22) by an investigator who treated the participants in the SMT group but was not involved in the assessments. Immediately after receiving a message that a new participant had been included in the study, this investigator initiated and organised their treatment.

Each of the participants in the SMT and GE groups received 6 treatment sessions lasting 30–45 min, with 1–2 treatment sessions per week, resulting in a total intervention period of 3–7 weeks. The number and frequency of the treatments were aligned to the usual physical therapy care covered by the statutory health insurance in Germany. The participants were free to perform additional exercises (SMT or GE, respectively) at home without supervision of the investigators. In case the participants wished to do so, the therapists suggested the number, type and intensity of home exercises.

All of the SMT participants were treated by the same physical therapist in individual sessions. The physical therapist had a bachelor’s degree, 2 years of clinical experience and had received specific training on SMT exercises.

The GE participants were treated in groups of up to 3 participants. Physical therapists, sports scientists or physical therapy students with 3–24 months of clinical experience and specific training on the GE exercise programme provided these treatments.

### Sensorimotor treatment group

The SMT was based on the programmes developed by Wand et al. [52] and Wälti et al. [53]. These interventions have demonstrated to be effective in reducing pain and disability in patients with NSCLBP [52, 53]. The SMT was adapted to the specific symptoms of the patients with CI. Therefore, it was focussed on the physical training component with sensory (localisation and graphesthesia training) and motor (laterality recognition and motor control) retraining. While the sensory training was similar to the regimens used in previous studies, the motor training component specifically emphasised CI-related issues by addressing aberrant movements and movement patterns stressing the patients’ pain tissues. However, compared to Wand et al. [52] and Wälti et al. [53], we focused less on the educational training component because we assumed that CI disorders mainly require increasing a patient’s level of control by training their movement patterns.

Both the sensory and the sensorimotor training components were offered at three difficulty levels with several options for variation and progression in each level (Table 1). The SMT therapist selected the exercises and tailored the dose based on the previous and actual performance of each participant with the aim of creating a moderate-to-high level of difficulty and ensuring that each participant was well focused. The decision on when to proceed to the next level was based on the previous and actual performance of each individual participant. While no objective criterion was used to determine when to proceed to the next level, the participants were usually trained at the same level for no more than two sessions before the difficulty level was increased.

**Table 1:** Exercise components included in the SMT

	Sensory part	Sensorimotor part	Laterality recognition training [54]
Sessions 1 + 2	Localisation training (left/right); detection of the number and localisation of points (starting distance: two-point discrimination threshold) Progression: ↑ number of points; ↑ speed; ↓ distance between points	Pelvic tilt exercises; finding a middle position of the lower back in different postures (comparable to movement control tests) [26] Progression: Starting position (easy to hard)	Recognition of the position of trunks pictured on a smartphone Progression: ↑ speed of the image sequence; ↑ number of images
Sessions 3 + 4	Identification of numbers; simple arithmetic tasks (addition and subtraction of numbers from 0 to 9) Progression: ↓ size of numbers; varying the orientation; ↑ speed	Movement control exercises comparable to movement control tests [26]; finding and maintaining a middle position of the lower back while performing simple movements of the trunk or extremities. Progression: Starting position; performing additional movements	As above. Individual progression based on actual performance
Sessions 5 + 6	Identification of letters; recognition of three-letter words. Progression: ↓ font size; varying the orientation; ↑ speed	As above, but with additional complex movements of the trunk or extremities; transfer to functional activities related to the patient’s symptoms. Progression: Complexity of starting position; use of additional weights	As above. Individual progression based on actual performance; ↑ complexity of images

The sensory discrimination training component targeted tactile acuity of the lower back through graphaesthesia training that used numbers and letters. The sensorimotor training component involved the precise execution of selective movements or holding a body position with a focus on the lower back. Often, the participants were asked to find and maintain a middle position of the lower back while moving their extremities or trunk, based on the motor control tests developed by Luomajoki et al. [26].

In addition, the SMT included a laterality recognition training component that used the “Recognise Back”® software (NOI Group) [54]. Participants were asked to differentiate between pictured rotation and the lateral bending directions of trunks in various difficulty levels. Data on laterality judgement accuracy and time exposure were collected. A detailed description of the SMT is presented in Table 1.

### General exercise group

The GE programme was used in a large multicentre randomised controlled trial and is well established in Germany [55]. It consists of four basic exercises (quadripedal position/prone bridging, rowing in a standing position, standing balance/knee bend and side support) with 12 levels of difficulty. Some levels of difficulty can be performed without additional tools, while other levels require unstable surface pads, additional weights, a ball, or other means of perturbation (Table 2).

**Table 2:** Exercises of the GE programme at difficulty levels 1, 6 and 12

Exercise 1: Quadripedal position/prone bridging		Exercise 2: Rowing in a standing position		Exercise 3: Standing balance/knee bend		Exercise 4: Side support	
Stable underground	Unstable underground	Stable underground	Unstable underground	Stable underground	Unstable underground	Stable underground	Unstable underground
1. with bending/ straightening of one leg	6. lean on foot and hand and reciprocally straighten one leg 12. push up and lift arm reciprocally	1. rowing in hip width stand with weight 6. single-arm rowing with trunk rotation in hip width stand on the forefoot with weight	12. single-arm rowing with trunk rotation in one leg stand on the forefoot with weight	1. stand on both legs and change from forefoot to toes stand 6. one leg stand on the forefoot with hip abduction and extension of the swinging leg	12. one leg knee bend with weight	1. knee on the ground, pelvis up and down 6. straighten legs and pelvis up and down; rolling a ball	12. straighten legs and pelvis up and down; bring upper leg and arm in front of the body together; straighten and bounce a ball

The numbers present 3 out of 12 selected stages of difficulty for each exercise: 1 (easiest level), 6 and 12 (hardest level)

In every session, 3 sets of 15 repetitions of each exercise were performed by each participant. In the first session, the level was selected based on a patient’s performance in the quadripedal position/prone bridging exercise. The level was reviewed and adjusted in the third and the fifth sessions.

### Feasibility, outcome measures, and follow-up

The feasibility criteria included the study design, assessments, interventions, magnitudes of effects, and costs (Table 3). The total costs were assessed by assuming standard hourly rates for the physiotherapy research assistants performing the recruitment and assessments and for the physiotherapists guiding the interventions, as well as considering the costs of the study materials. Our feasibility study was executed under a master’s thesis project, and it was part of the usual activities of the university staff involved. Therefore, the aim was to calculate the costs for a larger externally funded study.

**Table 3:** Criteria for feasibility of the pilot randomised controlled trial

	Explanation	Operationalisation and unit
Study design		
Recruitment rate	Relation between interested persons, eligible persons and study participants	n study participants / n interested persons [%]; n study participants / n eligible persons [%]
Recruitment period	Time expenditure for recruitment of study participants	Time [weeks]
Randomisation	Comparability of the randomised groups	Differences in primary outcomes between intervention- and control group at the beginning of the study [*p*-value]
Blinding	Practicability and success rate of the blinding of the assessor	Proportion of the participants, who inadvertently reveal their group assignment to the assessor [%]
Dropout rate	Drop-outs	n study participants who have not completed the study according to the protocol/ n study participants [%]
Assessments		
Safety	Occurrence of adverse events/ complications	Absolute n of participants who had adverse events or showed aggravated symptoms related to the assessments
Duration	Duration of the test battery including the questionnaires in minutes	Time [min]
Acceptance	Participation in assessments and questionnaires	n of declined assessments or questionnaires/ n study participants [%]
Interpretability/Completeness	Occurrence of floor and ceiling effects	≥ 15% of study participants cannot perform an assessment; ≥ 15% of the study participants have the highest or lowest value of the scale
	Missing values in questionnaires or assessments	n missing values / n Items [%]. Critical if the median of missing items in a questionnaire or assessment is ≥10%.
Intervention		
Extent of the treatment	Frequency	sessions per week per study participant
	Duration	Median duration of one session [min]
Feasibility of the exercises	Refusal rate and estimation of the feasibility by the therapist	n exercises refused/ n exercises offered [%]; structured rating of the feasibility by the therapist
Acceptance of the intervention	Subjective estimation of the study participants	Standardised personal rating by the participants
Adverse events	Adverse events or complications, e.g. pain, loss of function, and consultation of a physician	Overall n of adverse events (mild/moderate/severe) which are not, unlikely, possibly, probably or definitely related to the intervention
Costs		
Staff	Training: investigation of the outcomes	Total Duration [min]; cost [€]
	Training: execution of the intervention	Total Duration [min]; cost [€]
	Screening and examination of the inclusion and exclusion criteria	Total Duration [min]; cost [€]
	Investigation of the outcomes	Total Duration [min]; cost [€]
	Execution of the intervention	Total Duration [min]; cost [€]
	Additional contact to the study staff initiated by the participants (questions and wishes)	Total Duration [min]; cost [€]
Structure	Measurement instruments (material costs, license costs, software for the analysis)	Cost [€]
	Equipment for the exercises	Cost [€]
	Ethics proposal	Cost [€]
	Registration of the study	Cost [€]
	Recruitment material (Flyer)	Cost [€]

We observed and documented any adverse effects in every treatment session. Moreover, we asked the standard question, “Have you observed any changes in your symptoms since you started this treatment?”. Informed by Lineberry et al. and Kelly et al. [56, 57], the authors consented to classify document adverse events as follows:

1. Minor: The issue can be solved or treated by providing a short break. Consultation with a physician is not necessary. The assessment/exercise session is continued on the same day (e.g. slight muscle pain with remission within minutes).
2. Moderate: The assessment/exercise session must be aborted. Consultation with a physician is not necessary. The assessment/exercise session is continued on another day (e.g. acute, moderate joint pain with remission on the following day).
3. Severe: The assessment/exercise session must be aborted. Consultation with a physician is necessary. The assessment/exercise session may be continued on another day or the participant may not be able to continue the assessment/exercise session (e.g., muscle strain).

In case it was unclear whether an event was moderate or severe, a physician was consulted.

To estimate the effects, primary outcome measures were selected by using the core outcome set (COS) developed by Chiarotto et al. [58]. The primary outcomes included pain severity and interference (Brief Pain Inventory, BPI) [59, 60], physical function (Roland-Morris Disability Questionnaire, RMDQ [61, 62], and Oswestry Disability Index, ODI [63–65]), and health-related quality of life (Short-Form 36, SF-36) [66–68]. The secondary outcome measures were movement control (movement control test battery, MCTB) [27, 69], tactile acuity (two-point discrimination threshold, TPDT) [28, 70, 71], fear-avoidance beliefs (Fear Avoidance Belief Questionnaire, FABQ) [72, 73] and anxiety and depression (Hospital Anxiety and Depression Scale, HADS) [74, 75].

The outcome measures were assessed twice at baseline 1 week apart (t1a and t1b) to estimate the test-retest reliability (methodological and biological variation) over time and once more after completion of the intervention (t2). All measurements were performed by the same blinded assessor who had frequently practised the TPDT and MCTB measurements before the start of this study.

### Blinding

The assessor was blinded to the treatment allocation. Due to the nature of the interventions, it was impossible to blind the therapists or patients. The participants were asked not to mention their group allocation during follow-up assessments to avoid un-blinding the assessor.

### Statistical analysis

The statistical analysis was conducted on an intention-to-treat basis. In cases of missing data, the ‘last observation carried forward’ procedure was used. We used IBM SPSS Version 22 for performing all of the analyses. Both on-site investigators performed the statistical analyses and were not blinded to treatment allocation.

The feasibility outcomes were reported descriptively and narratively.

For the primary and secondary clinical outcomes, mean (standard deviation, SD) was reported for the continuous data, medians (interquartile range, IQR) for the ordinal outcomes, and raw count (number, %) for the nominal data.

At baseline, in the entire study population, the relative test-retest (t1a and t1b) reliability of the primary and secondary outcomes was assessed using the intraclass-correlation coefficient (ICC) model 2.1 (two-way random effects model) [76]. The ICC was calculated by dividing the systematic differences between the “true” scores of the patients by the error variance, which consists of the systematic differences between the true scores of the patients, variance due to systematic differences between the two measurements, and residual variance [76]. ICC values ≥0.7 were considered acceptable for group comparisons over time [77].

If normal distribution and interval scaling applied, the intergroup differences at baseline were tested using the t-test for independent samples between t1a and t2. Most of the intergroup and intragroup differences (BPI, RMDQ, ODI, SF-36, TPDT, MCTB, HADS, FABQ) were tested using the Mann-Whitney-U-test or Wilcoxon signed-rank test. For the outcomes with data on a nominal scale, the chi^2^-test was used. The level of statistical significance was set to *p* < 0.05 [78]. No alpha-correction was applied due to the exploratory nature of this pilot study.

In the case of a significant difference, we additionally calculated the effect size r ($r=\frac{Z}{\surd n}$). Effect sizes > 0.1 were regarded as small, those > 0.3 as moderate, and those > 0.5 as large [79].

Furthermore, we calculated the sample size needed to detect differences in pain severity between the SMT and the GE groups of patients with NSCLBP with 80% power.

### Funding

This study was not funded externally.

## Results

**Fig. 2:**
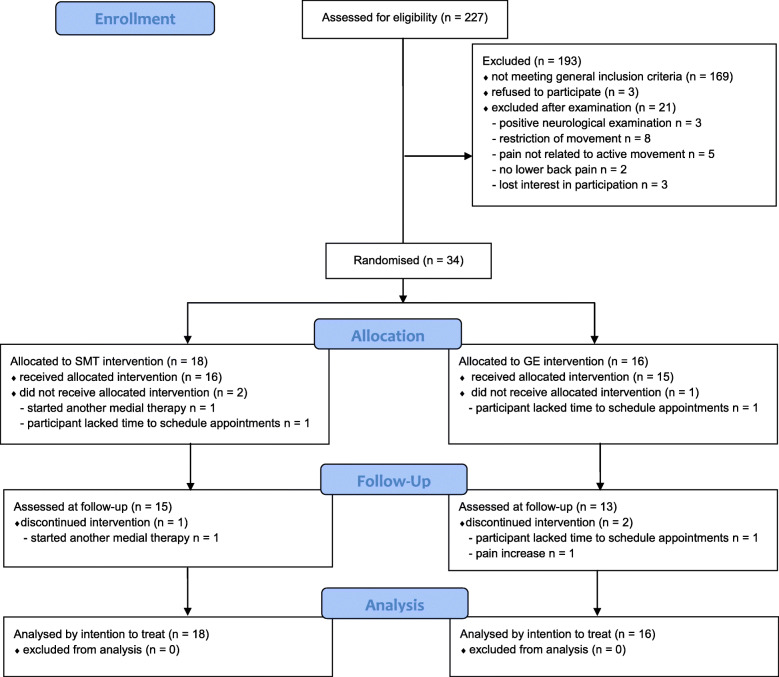
CONSORT flow diagram of study enrolment, intervention allocation, follow-up, and data analysis

Between September 2016 and March 2017, 227 patients were screened for inclusion, of which 34 participants were randomised into the SMT and GE groups. Three participants in each group did not complete their participation in the study as planned, resulting in a dropout rate of 16.7 and 18.6%, respectively (Fig. 2). One of these participants started a medical intervention and was not aware that this would necessitate their exclusion from the study (SMT group). Two participants (one in each group) ended their participation because of time constraints. Two participants in the GE group quit during intervention period, one of whom cited organisational reasons and the other cited increased pain severity because of the intervention. One participant in the SMT group terminated the intervention because they commenced a planned medical intervention without connection to the SMT intervention.

At baseline (t1a), the patients who later dropped out were older (*p* = 0.021) and showed higher pain severity (*p* = 0.010) than the patients who completed the study.

### Participant characteristics

Most of the participants were female (78%) and reported having back pain for 14–63 months. While the 18 participants randomised into the SMT group were slightly older, the characteristics did not differ significantly from those participants in the GE group (Table 4). The baseline scores of most participants indicated mild-to-moderate pain severity, pain interference, and limitation of function (Table 4). These scores and further baseline outcome scores did not differ significantly between the SMT and the GE groups, with *p*-values ranging from 0.12 to 1.00 (*p*-values of the baseline intergroup comparisons are not shown in the table). No participant needed to be excluded due to an RMDQ Score > 18.

**Table 4:** Baseline characteristics of *n* = 34 patients with NSCLBP and CI

Characteristics	Sensorimotor training group, *n* = 18	General exercise group, *n* = 16	chi^2^ (df) p	t (df) p	z_U_ p
Sex					
female n (%)	14 (78%)	12 (75%)	0.036 (1) 1.00		
male n (%)	4 (22%)	4 (25%)			
Height (m) mean (SD)	1.72 (0.08)	1.72 (0.09)		0.049 (26) 0.96	
Weight (kg) mean (SD)	75.9 (15.3)	76.4 (20.6)		−0.065 (26) 0.95	
Age^a^ (years) median (IQR)	51 (26–59)	37 (24–50)			−1.398. 0.16
Low back pain duration^a^ (months) median (IQR)	36 (14–54)	36 (17–63)			−0.381. 0.71

*N* Number of patients, *SD* Standard deviation, *M* Median, *IQR* Interquartile range, *df* Degrees of freedom, ^a^ Non-normal distribution

### Feasibility

Two investigators worked for 29 weeks to contact 227 interested patients and finally included 34 participants. With only one exception, all participants were able to perform the complete range of physical tests and fill out all questionnaires. The participants perceived the duration and character of the study assessments as adequate. We did not record the exact duration of each assessment session, but the assessor reported that one session took approximately 50–70 min. There were no floor and ceiling effects for any of the reported outcome measures.

Both the SMT and the GE interventions were well accepted. Most of the sessions were performed as scheduled. The actual duration of the SMT and GE sessions was 40 min, and according to the therapists, the time was sufficient to complete the scheduled exercises. Two adverse events occurred, one of which was likely triggered by the GE intervention while the other was likely not triggered by the SMT intervention. Assuming hourly pay rates of €22.30 for the physiotherapy research assistants performing the recruitment and the assessments and €16.00 for the physiotherapists guiding the interventions, the total cost of this study was €10,918, which translates into a cost of €321 per participant (Table 5).

**Table 5:** Feasibility of the pilot randomised controlled trial for determining the effects of a SMT programme

**Study design**	
Recruitment rate	34 study participants / 227 interested persons [15.0%] 34 study participants / 37 eligible persons [91.9%]
Recruitment period	29 weeks
Randomisation	No significant baseline differences between SMT and GE group in terms of the primary outcomes (Table 5)
Blinding	1 study participant inadvertently revealed group assignment to the assessor [2.9%]
Dropout rate	6 dropouts/ 34 study participants [17.7%]
**Assessments**	
Safety	1 participant reported an immediate but temporary increase in pain, likely related to a movement control test [2.9%]
Duration	50–70 min for each assessment (t1a, t1b, t2)
Acceptance	0 absolute; 0 declined investigation or questionnaires among 34 study participants [0%]
Completeness and interpretability	1 study participant (2.9%) was unable to undergo an assessment (one test in the movement control test battery); ≥15% of the study participants achieved the highest or lowest value on the scale BPI: t1a *n* = 0, t1b *n* = 0, t2 *n* = 1 (lowest value) RMDQ: t1a *n* = 0, t1b *n* = 2 (lowest value), t2 *n* = 4 (lowest value) ODI: t1a *n* = 0, t1b *n* = 1 (highest value), t2 *n* = 0 SF-36: t1a *n* = 0, t1b *n* = 0, t2 *n* = 0 HADS: t1a *n* = 0, t1b *n* = 1 (lowest value), t2 *n* = 3 (lowest value) FABQ: t1a *n* = 0, t1b *n* = 0, t2 *n* = 0
	n missing values / n Items [%] BPI: *n* = 8 missing values / *n* = 15 Items × 34 = 510 (1.6%)RMDQ: *n* = 0 missing values / *n* = 24 Items × 34 = 816 (0%) ODI: *n* = 0 missing values / *n* = 10 Items × 34 = 340 (0%) SF-36: *n* = 0 missing values / *n* = 36 Items × 34 = 1224 (0%) FABQ: *n* = 0 missing values / *n* = 16 Items × 34 = 544 (0%) HADS: *n* = 1 missing values / *n* = 14 Items × 34 = 476 (0.2%)
**Interventions**	
Treatment dose	SMT group: Average frequency of 1.5 sessions per week (total intervention period of 4 weeks) GE group: Average frequency of 2 sessions per week (total intervention period of 3 weeks)
	Median duration of one session: 40 min (both SMT and GE group)
Feasibility of the exercises in the treatment groups	SMT group: *n* = 1 refused exercise / *n* = 6 offered exercises (16.7%) related to movement control in one session for 1 study participant GE group: *n* = 1 refused exercise / *n* = 8 offered exercises (12.5%) in one session for 3 study participants
Acceptance of the interventions	All participants in the SMT group exhibited and mentioned high acceptance for all the exercises. High acceptance was recorded for most exercises in the GE group. Side support was mentioned as being too difficult by some participants, and it introduced difficulties in the grading of the other exercises
Adverse events	*n* = 1 likely triggered by the GE program (moderate); *n* = 1 likely not triggered by the SMT program (minor)
**Costs (including employer’s contribution)**	
Staff	
Training: investigation of the outcomes	Total duration: 240 min; cost for two physical therapists: [€160]
Training: execution of the intervention	Intervention group – total duration: 420 min; cost for two physical therapists [€280] Control group – total duration: 420 min; cost for two physical therapists [€280]
Screening and examination of the in- and exclusion criteria	Telephone Screening: 10–20 min for each patient (*n* = 227); physical examination: 45–90 min for each patient (*n* = 55) Total duration [3405 + 4400 = 7805 min]; cost for one B.Sc. Physical Therapist [€3250]
Investigation of the outcomes	Duration of approximately 60 min for each measurement time point and three time points for each participant (*n* = 34) resulting in a total duration of 6120 min; cost for a M.Sc. Physical Therapist [€2850]
Execution of the intervention	Each intervention in both groups lasted 30–45 min (average of 38 min), and 6 sessions were conducted for each patient (*n* = 34) resulting in a total duration of 7752 min; cost for SMT intervention B.Sc. Physical Therapists [€2580]; cost for GE intervention (groups with 2–3 patients) [€825]
Contacts to the study staff initiated by the participants (questions and wishes)	Total duration: Approximately 240 min; cost for a B.Sc. Physical Therapist [€80]
**Structure**	
Measurement instruments (material costs, license costs, software for the analysis)	SF-36100 questionnaires [€82], HADS 100 questionnaires [€168], SPSS software pro rata [€100], and other material costs [€50]
Equipment for the exercises	Therapy Table pro rata [€100]
Ethics proposal	Fee [€300]; cost for a M.Sc. Physical Therapist for a duration of 900 min [€334]
Registration of the study (DRKS)	Total duration: 900 min, cost for a M.Sc. Physical Therapist [€334]
Recruitment material (Flyer, newspaper advertisement)	Flyer [€50]

*BPI* Brief Pain Inventory, *FABQ* Fear Avoidance and Beliefs Questionnaire, *GE* General exercise programme, *HADS* Hospital Anxiety and Depression Scale, *ODI* Oswestry Disability Index, *RMDQ* Roland Morris Disability Questionnaire, *SF-36* Short-Form 36, *SMT* Specific sensorimotor treatment

### Adverse events

One patient from the SMT group experienced a slight progression of symptoms for a duration of 2 days. The patient did not ascribe this progression to the intervention because the progression was first recognised 2 days after the fourth SMT session, and it clearly occurred because of an unusual movement during her everyday life. We classified this adverse event as minor and considered it unlikely to be related to the SMT intervention.

One patient from the GE group reported a considerable increase in pain directly after each session. The patient aborted the intervention after the third session because of this reason. We classified this adverse event as moderate and considered it likely to be related to the GE intervention.

No other patient from either the SMT or the GE group reported further adverse events. No participant died over the course of the study.

### Test-retest reliability of clinical outcome measures at baseline

The ICCs denoting the test-retest reliability of the primary and secondary outcomes based on the data of all the participants who completed both baseline measurements t1a and t1b (*n* = 32) ranged from 0.85 (BPI Intensity) to 0.94 (SF-36 psychological component) and from 0.72 (FABQ Activity) to 0.98 (HADS Anxiety), respectively (Table 6).

**Table 6:** Magnitude of effects of SMT and GE intervention on primary and secondary outcomes

	Median (IQR) t1a	Median (IQR) t1b	Median (IQR) t2	t1a vs. t1b (ICC)	Intragroup comparison t1a vs t2 p (r)	Intragroup Δt2-t1a median	Intergroup comparison of intragroup Δt2-t1a (p)
BPI Intensity							
SMT	3.50 (1.75–4.13)	3.00 (1.25–5.13)	1.13 (0.50–3.44)	0.85	0.01* (0.63)	-1.25	0.26
GE	3.00 (2.19–4.19)	3.75 (1.50–3.94)	2.00 (1.81–3.00)		0.07	-0.88	
BPI Interference							
SMT	1.93 (0.64–3.46)	1.57 (0.39–4.11)	0.36 (0.11–2.21)	0.90	≤0.001* (0.83)	-0.86	0.25
GE	1.50 (0.89–2.82)	1.43 (0.64–2.39)	0.93 (0.29–2.61)		0.17	-0.50	
RMDQ							
SMT	4.50 (2.00–8.75)	6.50 (2.75–10.25)	3.50 (1.00–7.00)	0.89	0.803	0.00	0.64
GE	5.00 (1.50–6.00)	4.00 (2.00–5.00)	3.00 (2.00–4.75)		0.03* (0.53)	-1.00	
ODI							
SMT	22.00 (9.50–30.50)	22.00 (13.50–32.00)	11.00 (6.00–26.50)	0.92	0.03* (0.51)	-3.00	0.97
GE	20.00 (12.00–26.67)	17.89 (10.50–22.00)	16.00 (10.50–21.67)		0.03* (0.53)	-4.00	
SF-36 PC							
SMT	38.62 (32.34–47.65)	38.98 (31.56–48.11)	46.43 (39.83–52.29)	0.91	0.01* (−0.67)	+6.47	0.11
GE	45.47 (37.18–48.20)	45.64 (37.40–52.98)	47.83 (43.70–52.41)		0.15	+3.09	
SF-36 MC							
SMT	50.51 (45.07–56.34)	53.51 (37.39–57.11)	55.40 (36.24–59.46)	0.94	0.94	+1.02	0.88
GE	54.09 (44.11–58.72)	54.20 (47.70–59.23)	52.77 (48.62–58.52)		0.27	+0.66	
2PD [mm]							
SMT	64.38 (48.75–76.88)	50.63 (46.88–63.75)	43.13 (35.00–66.56)	0.73	0.002* (0.75)	-13.13	0.09
GE	60.63 (41.88–68.44)	58.75 (43.75–68.13)	53.13 (39.38–66.56)		0.20	-3.13	
MCT [number of positive tests]							
SMT	4.00 (3.00–4.25)	4.00 (3.75–4.00)	1.50 (1.00–3.00)	0.85	≤0.001* (0.86)	-2.00	0.05*
GE	4.00 (3.00–5.00)	4.00 (3.25–5.00)	3.50 (2.00–4.75)		0.05	-1.00	
HADS A							
SMT	6.50 (3.00–10.25)	6.00 (2.75–11.00)	4.50 (2.75–9.25)	0.98	0.04* (0.50)	-1.00	0.67
GE	5.50 (2.00–8.75)	4.50 (2.00–8.00)	4.50 (2.00–8.00)		0.10	-1.00	
HADS D							
SMT	4.00 (1.00–6.00)	3.00 (1.00–5.25)	2.00 (1.00–5.00)	0.97	0.04* (0.50)	0.00	1.00
GE	2.50 (1.00–4.50)	2.00 (1.00–4.75)	1.00 (0.25–5.75)		0.09	-1.00	
FABQ Activity							
SMT	12.00 (10.75–15.25)	11.50 (8.75–14.50)	10.50 (6.75–12.00)	0.72	0.02* (0.53)	-1.50	0.44
GE	9.50 (6.25–14.50)	10.50 (5.75–16.00)	10.00 (5.50–14.75)		0.68	-1.50	
FABQ Work							
SMT	10.50 (2.00–18.00)	12.50 (3.75–21.25)	9.00 (2.75–19.00)	0.87	0.50	0.00	0.07
GE	8.50 (2.00–19.25)	10.00 (1.25–15.75)	8.50 (1.25–12.00)		0.02* (0.57)	-2.00	

*A* Anxiety, *BPI* Brief Pain Inventory, *D* Depression, *FABQ* Fear Avoidance and Beliefs Questionnaire, *GE* General exercise programme, *HADS* Hospital Anxiety and Depression Scale, *ICC* Intraclass-correlation coefficient, *MC* Mental component, *MCT* Movement control tests, *ODI* Oswestry Disability Index, *PC* Physical component, *RMDQ* Roland Morris Disability Questionnaire, *SF-36* Short-Form 36, *SMT* Specific sensorimotor treatment, *t1a* first measurement point, *t1b* second measurement point, *t2* follow-up measurement, *2PD* two-point discrimination

### Efficacy: primary and secondary outcomes

**Fig. 3:**
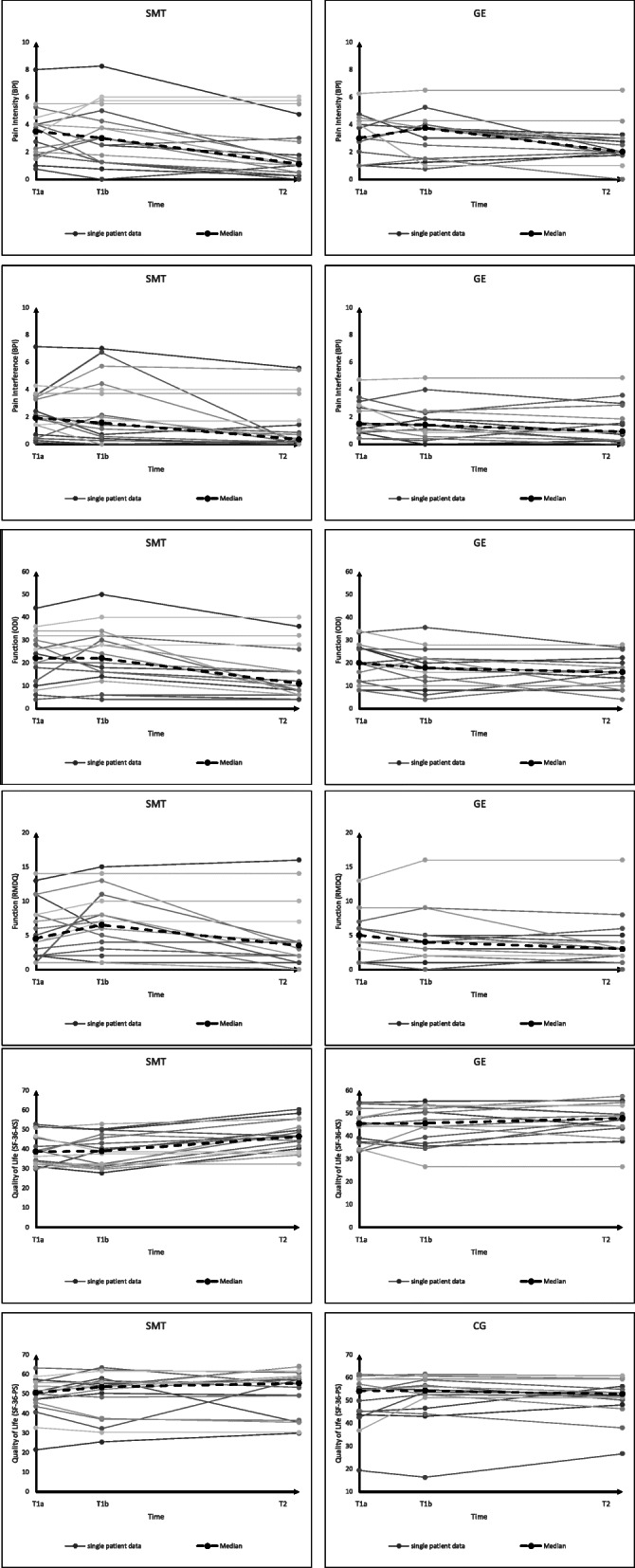
Individual values and median of pain, function and quality of life

In the SMT group, when the values at t2 (post) were compared with those at t1a (baseline), 5 of the 6 primary outcomes and 5 of the 6 secondary outcomes improved significantly, respectively. In the GE group, when the values at t2 were compared with those at t1a, 2 of the 6 primary outcomes and 1 of the 6 secondary outcomes improved significantly, respectively (Fig. 3, Table 6).

Movement control was the only outcome that exhibited a significant intergroup difference.

### Sample size calculation

To ensure the discovery of differences in pain severity (BPI) between a SMT and a GE programme for patients with NSCLBP and CI with 80% power, future studies must include at least 110 patients (55 in each group).

## Discussion

### Major findings

Overall, the components of this study, including both treatment programmes and the assessments, seemed feasible. The duration and nature of the assessments were perceived as adequate by the participants. The ICCs of all of the primary and secondary outcome assessments were ≥ 0.7, indicating that these assessments can be used in a larger study. Both the SMT and the GE interventions were well accepted in terms of their durations and contents. The total cost per participant, including those of the assessments and the interventions, was €321.

In the SMT group, one adverse event occurred, which was estimated to be minor and considered unlikely to be related to the SMT intervention. In the GE group, one adverse event occurred, which was estimated to be moderate and considered likely to be related to the GE intervention.

In terms of the magnitudes of the clinical effects, according to the within-group changes, the SMT programme exhibited potential for reducing pain, improving the physical component of quality of life, and improving movement control among patients with NSCLBP and CI. There were no differences between the SMT and the GE groups at t2, except for movement control.

### Clinical relevance and importance of the findings

The differences between the within-group changes in physical function of the patients in the SMT and the GE groups in our study were not clinically relevant. In the SMT group, pain severity decreased by 35% between t1a and t2. Changes of more than 30% in pain and function are considered clinically relevant [80]. Information about the minimal clinically detectable change of the motor control tests (MCT) is not available. It was demonstrated that the healthy participants had 1 positive MCT out of 6 on average. By contrast, patients with LBP had 3 positive tests on average [27]. At t2, the patients in the SMT group had a median of 1.5 (IQR = 1.0–3.0) positive tests in comparison to a median of 4 positive tests (IQR = 3.0–4.3) at t1a. Therefore, the results of the SMT group were similar to those of the healthy controls at t2. However, this inconsistency might be induced by learning effects due to the similarity of the test battery and the SMT.

The present study focussed on feasibility and was, therefore, not designed and powered to draw solid conclusions about clinical significance and relevance. Even so, it would be surprising if future larger studies find a clinically large magnitude of differences between SMT and GE. Instead of comparing SMT versus GE in a larger study, it might be more useful to compare a combination of GE and SMT versus GE alone.

### Implications of the findings for clinicians and policymakers

If the current results are reproduced in a larger study, SMT programmes can be considered as one part of an appropriate treatment approach for patients with NSCLBP and CI. Even so, SMT would serve as an addition to, rather than a replacement for the existing approaches.

However, the current health supply scenario in Germany does not allow for a complete physical examination, as we conducted in this study. The average duration of 60 min needed to conduct a thorough physical examination exceeds the typical time available to physical therapists in Germany. We suppose that this time is necessary for achieving a correct classification when using the classification system proposed by O’Sullivan.

In addition, there are barriers to implementing the treatment applied in our study. The common treatment time per session was 40 min for both groups in our study. This represents a huge discrepancy with the current situation in Germany, where the usual session lasts for approximately 20 min. A potential solution for this discrepancy could be the inclusion of parts of the treatment as home exercises. Especially, the motor control exercises and the laterality recognition training have high potential for implementation as a home-based training regimen.

Because motor learning plays a key role in the impact of the SMT programme, we hypothesise that a greater number of sessions is needed to achieve greater improvements in all of the outcomes.

Overall, the SMT programme should be tested in future studies with greater numbers of treatment sessions and longer intervention periods, possibly as an addition to GE. To investigate dose-response relationships, future studies should consider the effects of the intervention over different treatment lengths.

### Comparison of results with other studies

Wälti et al. [53] used a similar treatment in their study, albeit in a population without subgroup classification. They found significant group differences in terms of pain severity (*p* = 0.03) and TPDT (*p* = 0.02). Unlike the current study, they reported no significant differences in movement control, possibly because the intervention was less tailored to their non-classified patients.

Gutknecht et al. [81] investigated a similar sensorimotor treatment that included graphaesthesia training in a comparable population. They, too, used the classification system proposed by O’Sullivan. No significant differences were found in comparison to a group treated with motor control training only.

Overall, because of our small sample size and pilot character, these comparisons should be viewed cautiously.

### Strengths and limitations

Initially, the participants exhibited comparatively small restrictions in pain (BPI) and disability (RMDQ and ODI). The treatment effects may be different in patients with more severe back pain.

Programme adherence was satisfactory in both groups (dropout rate 17.7%), and it was consistent with that in comparable studies on patients with NSCLBP [53].

Although we made every effort to select the best available measurement instruments to cover the outcomes, some instruments were viewed critically [82], and psychometric properties varied or were unclear. Especially, the structural validity of the measures for physical functioning in patients with CLBP were viewed problematic [82]. However, our ICCs (t1a and t1b) indicated good test-retest reliability.

While we carefully tested the patients for eligibility and strictly adhered to published classification methods, we cannot rule out the possibility that patients who had mixed classification and symptoms, or did even not have a CI, were included.

Because SMT is more complex to administer and there is no standard programme, the patients in the SMT group were treated by a therapist with more occupational back pain experience than that of the therapists who treated the patients in the GE group. This setting is consistent with the actual healthcare situation in Germany, where providers of individual therapy often differ from providers of group therapy. A more competent therapist (or patients’ perception thereof), and the higher levels of attention given to the SMT participants as a natural consequence of receiving individual therapy might have introduced a small bias among patients to favour SMT. Meanwhile, the GE intervention in the group may have been perceived as more enjoyable. Furthermore, home exercises were not monitored in this study. In future interventions, the type and extent of home exercises should be explicitly explained to patients in both groups and thoroughly monitored to prevent differences between the groups.

Another limitation is that long-term effects were not evaluated in this study. Furthermore, the lack of a do-nothing control group and of an intervention group with both GE and SMT should be considered a limitation of this study. The natural course of the disease is extremely important especially for NSCLBP, and must be considered when interpreting the effects of an intervention [2, 3, 5]. Although the participants in both groups received exactly the same information about the expected effects of their respective treatments, the participants’ expectations and placebo effects may certainly have played a role, and we might examine them in subsequent studies.

The current guidelines emphasize the importance of multimodal treatment with a biopsychosocial approach [8]. The SMT programme does not fully address this recommendation.

One strength of this study was the fact that the assessor was blinded to the group allocation and baseline data. Furthermore, there was only one assessor. This constitutes an additional strength of the present study, because some measurement methods, namely MCT and TPDT, have a relatively high intratester-reliability (as compared to their intertester-reliability).

The treatment for each patient was selected based on the classification result, which allowed for the delivery of more personalised treatments.

The outcome domains and the assessments applied were selected based on a COS developed by Chiarotto et al. [58]. In future studies, a more recently published COS [83] can be used as the basis for selecting assessments. These assessments are slightly different compared to the assessments applied in this study. Consensus has currently been reached on the use of ODI version 2.1a or the 24-item RMDQ for the measurement of physical function, on the numeric rating scale for pain severity and on the SF-12 or the 10-item PROMIS Global Health form as a scale for health-related quality of life [83]. Additionally, we would rather recommend an RMDQ score of > 20 as an exclusion criterion for future studies based on relevant literature [51].

## Conclusion

The SMT approach proposed in this study was found to be feasible for the treatment of patients with NSCLBP and CI. If the promising results of our study are confirmed or combinations of GE and SMT are found to be superior to GE alone, specific SMT components should be considered for inclusion in the treatment of patients with NSCLBP and CI.

Future implications for research include conducting a larger study. To be able to detect differences in pain severity (BPI) between an SMT and a GE programme for patients with NSCLBP and CI with 80% power, future studies must include 110 patients (55 in each group). This sample size can be considered realisable in a rehabilitation research context. However, the question arises whether the clinically relatively small magnitude of effects would justify such a study. A study testing GE versus both GE and SMT might be more promising. The results would enable a more targeted and comprehensive management of patients with CNSLBP and CI.

## Data Availability

The data collected during study application is available from the corresponding author on reasonable request.

## Abbreviations

BPI: Brief Pain Inventory
CI: Control impairment
CLBP: Chronic low back pain
COS: Core outcome set
CRPS: Complex regional pain syndrome
FABQ: Fear Avoidance Belief Questionnaire
GE: General exercise
HADS: Hospital Anxiety and Depression Scale
ICC: Intraclass-correlation coefficient
IQR: Interquartile range
LBP: Low back pain
MCT: Movement control test
MCTB: Movement control test battery
NSCLBP: Nonspecific chronic low back pain
ODI: Oswestry Disability Index
RMDQ: Roland-Morris Disability Questionnaire
SD: Standard deviation
SF-36: Short-Form 36
SMT: Sensorimotor treatment
S1: Somatosensory cortex
TPDT: Two-point discrimination threshold
